# Synthetic Thymidine Analog Labeling without Misconceptions

**DOI:** 10.3390/cells11121888

**Published:** 2022-06-10

**Authors:** Anna Ivanova, Olesya Gruzova, Elizaveta Ermolaeva, Olga Astakhova, Sheed Itaman, Grigori Enikolopov, Alexander Lazutkin

**Affiliations:** 1 Institute of Higher Nervous Activity and Neurophysiology, Russian Academy of Science, Moscow 117485, Russia; anivis33@gmail.com (A.I.); olesya.gruzova@mail.ru (O.G.); eliza.ermolaeva@gmail.com (E.E.); olga020302@gmail.com (O.A.); 2 Institute for Advanced Brain Studies, Lomonosov Moscow State University, Moscow 119991, Russia; 3 Center for Developmental Genetics and Department of Anesthesiology, Stony Brook University, Stony Brook, NY 11794, USA; Sheed.Itaman@stonybrook.edu; 4 Graduate Program in Neurobiology, Stony Brook University, Stony Brook, NY 11794, USA

**Keywords:** neurogenesis, thymidine analog, EdU, BrdU, DNA labeling, cell division, proliferation, neural stem cell

## Abstract

Tagging proliferating cells with thymidine analogs is an indispensable research tool; however, the issue of the potential in vivo cytotoxicity of these compounds remains unresolved. Here, we address these concerns by examining the effects of BrdU and EdU on adult hippocampal neurogenesis and EdU on the perinatal somatic development of mice. We show that, in a wide range of doses, EdU and BrdU label similar numbers of cells in the dentate gyrus shortly after administration. Furthermore, whereas the administration of EdU does not affect the division and survival of neural progenitor within 48 h after injection, it does affect cell survival, as evaluated 6 weeks later. We also show that a single injection of various doses of EdU on the first postnatal day does not lead to noticeable changes in a panel of morphometric criteria within the first week; however, higher doses of EdU adversely affect the subsequent somatic maturation and brain growth of the mouse pups. Our results indicate the potential caveats in labeling the replicating DNA using thymidine analogs and suggest guidelines for applying this approach.

## 1. Introduction

Marking proliferating cells by tagging their duplicating DNA with synthetic thymidine analogs has become a key approach in cell and developmental biology and neurobiology [1,2,3,4]. Although other widely applied markers of dividing cells, such as Ki67, PCNA, Mcm2, or phosphorylated histone H3, are highly useful, their precision and sensitivity are lower than those of the nucleotide-based analysis; moreover, unlike nucleotide tags, they cannot be used to trace the progeny of labeled cells [5]. The halogenated thymidine analog 5-bromo-2′-deoxyuridine (BrdU) is the most widely used analog for labeling duplicating DNA [1,2,3]. However, detecting incorporated BrdU-labeled residues with antibodies requires a lengthy procedure that is not always easily standardized for different cell and tissue types. In addition, in many cases, being able to mark select stages of the division cycle or trace cells’ progeny by using two or more DNA labels is highly advantageous; however, most of anti-BrdU antibodies cross-react with other’s halogenated nucleotides, such as 5-iodo-2′-deoxyuridine and 5-chloro-2′-deoxyuridine [2,6,7,8,9], thus complicating the interpretation of the results.

The terminal alkyne-bearing nucleotide 5-ethynyl-2′-deoxyuridine (EdU) has gradually become the analog of choice for both single- and multilabel experiments [2,4,7,8]. EdU residues incorporated in DNA can be readily identified through their click chemistry reaction with fluorescent azides, which are small-molecule compounds (unlike anti-BrdU antibodies) and, therefore, rapidly and efficiently penetrate tissue sections and entire organs [4,10]. Furthermore, the EdU detection protocol is fast and sensitive, and EdU can be combined with other markers of duplicated DNA and cell cycle [8,11,12]. However, several widely held assumptions limit the use of EdU in studies of neurogenesis: EdU is often assumed to be detrimental to the developing brain and have greater toxicity than BrdU; its bioavailability in different tissues has not been thoroughly studied; it is believed to work in a narrow concentration range, such that decreasing the EdU dose would decrease the number of labeled cells.

Here, we address those concerns by following the morphometric characteristics throughout the postnatal development in mice injected with various doses of EdU, as well as by quantitatively analyzing a neurogenic brain region in adult mice receiving a wide range of concentrations of EdU, BrdU, or their combinations. Our results suggest optimized experimental settings for labeling dividing cells and their progeny.

## 2. Materials and Methods

### 2.1. Animals and Treatments

Male C57Bl/6J mice (RRID:IMSR_JAX:000664, The Jackson Laboratory, Bar Harbor, ME, USA) were used to study the effects of thymidine analog administration on adult hippocampal neurogenesis. Adult males were group-housed at five or six animals per cage in standard cages (36 cm × 21 cm × 13.5 cm). To examine the immediate effects of EdU administration on cell proliferation, we injected 2-month-old males intraperitoneally (i.p.) with EdU (30540, Lumiprobe, Moscow, Russia) at doses of 20 (*n* = 6), 40 (*n* = 6), 80 (*n* = 6), or 123 mg/kg (*n* = 6) or equimolar doses of BrdU (B5002, Sigma-Aldrich, St. Louis, MO, USA) at 25 (*n* = 6), 50 (*n* = 6), 100 (*n* = 6), or 150 mg/kg (*n* = 6). In both cases, mice were deeply anesthetized with a mixture of Zoletil 100 (40 mg/kg, Virbac, Carros, France) and xylazine (5 mg/kg, Bioveta, Ivanovice na Hané, Czech) and euthanized 2 h after the nucleotide injection. Their brains were dissected, fixed in 4% paraformaldehyde (PFA) (158127, Sigma-Aldrich, St. Louis, MO, USA) overnight at 4 °C, and sectioned. The number of labeled cells in the dentate gyrus (DG) of the hippocampus was then determined. To examine the longer-term effects of EdU administration, we performed two types of experiments. In the first, 3-month-old males were injected i.p. with saline (0.9% NaCl, *n* = 6) or EdU at a dose of 40 (*n* = 6) or 123 mg/kg (*n* = 6); a total of 46 h after EdU injection, the mice received a single injection of BrdU at 50 mg/kg and were euthanized 2 h later. In the second experiment, 3-month-old C57Bl/6J males were injected i.p., either with EdU (40 (*n* = 5) or 123 mg/kg (*n* = 5)) or BrdU (50 (*n* = 4) or 150 mg/kg (*n* = 4)), and the number of labeled cells was determined 6 weeks after the injections.

C57Bl/6J pups of both sexes were used to study the developmental effects of EdU administration. For breeding, adult animals (one male and two or three females) were housed together in the colony room in standard 36 cm × 21 cm × 13.5 cm cages. Pregnant dams were housed individually from day 16 of gestation and inspected for births once per day at the same time of day. The presence of newborn pups was considered the date of birth and designated postnatal day 1 (PND1). Somatic maturation screening was applied from PND1 to PND17 to 34 pups of both sexes from four litters. The survival rates of the litters ranged from 86% to 100%. Rare dead animals were excluded from the subsequent analysis. On PND1, pups were weighed and individually tagged with a marker pen and received a subcutaneous (s.c.) injection of saline or EdU at appropriate concentrations and a volume of 10 µL per 1 g bodyweight. Newborn pups from different litters (7–10 pups per litter) were randomly distributed into four groups, denoted *SAL* (*n* = 10), *40* (EdU 40 mg/kg, *n* = 7), *60* (EdU 40 mg/kg, *n* = 8), and *123* (EdU 123 mg/kg, *n* = 6). On PND16, pups received one injection of BrdU (50 mg/kg). On PND17, mice were deeply anesthetized with a mixture of Zoletil 100 (40 mg/kg) and xylazine (5 mg/kg), and their brains were dissected, fixed in 4% PFA overnight at 4 °C, and rinsed with phosphate-buffered saline (PBS).

Before and during the experiment, all animals were housed under standard conditions at room temperature and a 12/12 h light-dark cycle, and they were maintained with food and water ad libitum. All experiments were approved by the Institutional Ethics Committee of Institute of Higher Nervous Activity and Neurophysiology, Russian Academy of Sciences (Protocol No. 10, 10 December 2012) and Stony Brook University IACUC; experiments were performed in accordance with the requirements of the Ministry of Health of the Russian Federation (Decree No. 267, 19 June 2003) and guidelines of the Declaration of Helsinki, as well as the National Institutes of Health Guide for the Care and Use of Laboratory Animals.

### 2.2. Somatic Analysis

To investigate the somatic development of pups injected with EdU, we used somatic tests from the extended version of Fox’s battery of neurotoxicity tests [13]. The screening included morphometric and somatic indices of general development, which indicate sensitivity to genetic, environmental, pharmacological, and other manipulations and serve as parameters of somatic and physical development [13,14,15,16,17].

Each day, a dam was removed from the home cage and placed in an individual clean cage with bedding. The home cage with the litter was transferred to the experimental room, and individual pups were examined. After analysis, individual pups’ ink marks were refreshed, and the dam was returned to the home cage. The somatic development test battery included the following measurements:

- *Bodyweight* (PND1–PND17). PND1 to PND8 pups were placed on the pan of an electronic balance (OHAUS Explorer Pro, Parsippany, NJ, USA), which was covered with paper. Starting from PND9, weighing was performed in a plastic container.
- *Milk in the stomach* (PND1–PND6). A pup was gently supported in supine position by the experimenter’s fingers. During the first week of life, the pup’s skin is transparent under the abdomen area, thus enabling evaluation of the presence of milk, which is visible as a white spot in the pup’s stomach area. Visual estimation of milk in the stomach was performed. A score of 1 was given if milk was present in the stomach, and a score of 0 was given if milk was absent.
- *Hair appearance* (PND1–PND4). Primary hair appearance was assessed by visual determination of fine hairs under contrasting lamplight. A score of 1 was given if the fine primary hairs were apparent, and a score of 0 was given if they were absent.
- *Auricle separation* (PND1–PND6). The separation of the auricles from each pup’s head was estimated visually. If auricles were not separated, the experimenter used a small wooden probe to gently separate the ears from the head. A score of 0 was given if both ears were not separated from the head. Separation of one or two ears was recorded as a score of 1 or 2, respectively.
- *Finger separation* (PND1–PND10) was determined with a wooden probe; separation was considered complete when all paw fingers were clearly separated. A score of 0 was assigned if no separation was observed between all fingers. Scores of 1 to 10 were assigned, depending on the number of separated fingers. Fore- and hindlimbs were evaluated separately.
- *Incisor teething* (PND10–PND14). The appearance of lower and upper incisors was visually evaluated. The absence of incisors was assigned a score of 0. The appearance of one and two incisors was scored as 1 and 2, respectively.
- *Complete fur covering* (PND10–PND15). The criterion for complete fur covering was the presence of a crest when a finger or a small stick was run along the ventral abdominal line. The presence of covering was scored as 1, and its absence was scored as 0.
- *Eye opening* (PND11–PND17). This parameter was assigned a score of 0 if the eyes were closed. For complete opening of one or two eyes, a score of 1 or 2, respectively, was given.
- *Brain weight* (PND17). Fixed brains were rinsed with PBS, dried with filter paper, and weighed on electronic balances. The spinal cord was cut off before weighting.

### 2.3. Staining

*Brain sections.* Sagittal 50 µm sections from a randomly selected hemisphere were prepared with a Leica VT1200S vibratome (Leica Biosystems, Wetzlar, Germany). The sections were subsequently collected in six wells of 24-well tissue culture plates, so that each section in the same well was positioned 300 μm apart from the next one in the brain. Sets from one well, containing an average of seven to nine slices, covering the entire DG, were used for staining and cell counting [18]. Sections were kept in PBS at 4 °C or cryoprotectant (PBS, ethylene glycol, and glycerol, 2:1:1, all from Sigma-Aldrich, St. Louis, MO, USA) at −20 °C until staining.

*EdU staining.* Sections were rinsed with PBS three times after cryoprotectant treatment and permeabilized with 2% Triton X-100 (Sigma-Aldrich, St. Louis, MO, USA) in PBS for 1 h at room temperature (RT). Then, the sections were stained through a click reaction in PBS containing 0.2% Triton X-100, 120 mM sodium ascorbate (Sigma-Aldrich, St. Louis, MO, USA), 6 mM CuSO_4_ (Sigma-Aldrich, St. Louis, MO, USA), and 10 µM AlexaFluor 555 Azide (A20012, Invitrogen, Waltham, MA, USA) for 20 min at RT in the dark. The click reaction was stopped by incubation with 0.1 M EDTA (Invitrogen, Waltham, MA, USA). After three washes with 0.2% Triton X-100 in PBS, sections were counterstained with Hoechst 33342 (1 µg/mL, H3570, Invitrogen, Waltham, MA, USA) for 15 min at RT in the dark and washed twice with PBS.

*BrdU staining.* Before immunolabeling for BrdU, tissue sections were treated for DNA denaturation by incubation in 2 N HCl (Sigma-Aldrich, St. Louis, MO, USA) for 30 min at 37 °C; then, they were neutralized with 0.1 M sodium borate (pH 8.5, Sigma-Aldrich, St. Louis, MO, USA). Sections were rinsed with PBS three times and incubated with blocking and permeabilization solution containing 2% Triton X-100 and 5% normal goat serum (NGS) (ab7481, Abcam, Cambridge, UK) in PBS for 1 h at RT. Incubation with rat anti-BrdU antibody (1:500, OBT0030G, AbD Serotec, Hercules, CA, USA) was performed in PBS, 0.2% Triton X-100, and 5% NGS overnight at 4 °C on a shaker. Then, sections were washed three times with 0.2% Triton X-100 in PBS. Incubation with goat anti-rat AlexaFluor 488 antibody (1:500, A11006, Invitrogen, Waltham, MA, USA) in PBS, 0.2% Triton X-100, and 5% NGS was performed for 2 h in the dark at RT. Hoechst 33342 (1 µg/mL) was added to the incubation solution for counterstaining.

*BrdU and EdU co-staining.* The brain sections were treated for DNA denaturation by incubation in 2 N HCl for 30 min at 37 °C and then neutralized with 0.1 M sodium borate (pH 8.5). Sections were rinsed with PBS three times and incubated with blocking and permeabilization solution containing 2% Triton X-100 and 5% NGS PBS for 1 h at RT. Incubation with mouse monoclonal anti-BrdU antibody (1:500, MoBU-1, 317902, BioLegend, San Diego, CA, USA) was performed in PBS, 0.2% Triton X-100, and 5% NGS overnight at 4 °C on a shaker. Then, the sections were washed three times with 0.2% Triton X-100 in PBS. Incubation with goat anti-mouse highly cross-adsorbed AlexaFluor 488 antibody (1:500, A11029, Invitrogen, Waltham, MA, USA) in PBS, 0.2% Triton X-100, and 5% NGS was performed for 2 h in the dark at RT. Hoechst 33342 (1 µg/mL) was added to the incubation solution for counterstaining. After three rounds of washing with 0.2% Triton X-100 in PBS, the sections were stained with a click reaction in PBS containing 0.2% Triton X-100, 120 mM sodium ascorbate, 6 mM CuSO_4_, and 10 µM AlexaFluor 555 azide for 20 min at RT in the dark. The click reaction was stopped by incubation with 0.1 M EDTA. Sections were washed three times with 0.2% Triton X-100 in PBS and twice with PBS.

*DCX and NeuN staining.* For triple-staining of EdU, NeuN, and DCX, a click reaction with AF488 azide (11830, Lumiprobe, Moscow, Russia) was first performed as described. The sections were then incubated with guinea pig anti-DCX antibody (1:2000, AB2253, Millipore, St. Louis, MO, USA) in PBS, 0.2% Triton X-100, and 5% NGS overnight at RT. After three rinses in 0.2% Triton X-100 in PBS, the sections were incubated with goat anti-guinea pig AlexaFluor 647 antibody (1:500, A21450, Invitrogen, Waltham, MA, USA) in PBS, 0.2% Triton X-100, and 5% NGS for 2 h in the dark at RT. After three rinses in 0.2% Triton X-100 in PBS, the sections were incubated overnight with mouse anti-NeuN AlexaFluor 555 conjugates (1:1000, MAB377A5, Millipore, St. Louis, MO, USA) in PBS, 0.2% Triton X-100, and 5% NGS in the dark at RT. For triple-staining of BrdU, NeuN, and DCX, DNA denaturation and permeabilization were performed as described above. Then, the sections were incubated with mouse monoclonal anti-BrdU antibody (1:500) and guinea pig anti-DCX antibody (1:2000) in PBS, 0.2% Triton X-100, and 5% NGS overnight at 4 °C. After three rinses in 0.2% Triton X-100 in PBS, the sections were incubated with goat anti-mouse AlexaFluor 488 antibody (1:500) and goat anti-guinea pig AlexaFluor 647 antibody (1:500) in PBS, 0.2% Triton X-100, and 5% NGS for 2 h in the dark at RT. After three rinses in 0.2% Triton X-100 in PBS, the sections were additionally incubated overnight with mouse anti-NeuN AlexaFluor 555 conjugates in PBS, 0.2% Triton X-100, and 5% NGS in the dark at RT. The sections were washed three times with 0.2% Triton X-100 in PBS and three times with PBS in the dark.

### 2.4. Image Analysis

Brain sections were imaged using a confocal laser scanning microscope Olympus FluoView FV1000 (Olympus, Tokyo, Japan) with a water-immersion 40× objective (NA 0.8). All images were counted using Imaris 7.6.4 software (RRID:SCR_007370, Bitplane, Belfast, UK). The number of EdU^+^ cells was also visually estimated using the Nikon Eclipse Ti microscope (Nikon, Tokyo, Japan) with 20× (NA 0.75) and 40× (NA 0.95) objectives. For brain cell analysis, the number of cells in the granule and subgranule cell layers of the DG was counted [9,18]. The counts of labeled cells from all sections of one brain were averaged, then normalized to the average number of sections from all animals in the experiment; then, the total number of cells per two hippocampi was counted by multiplying the normalized average count to 6 (number of sets) and 2 (number of hemispheres). Representative images were imported into Adobe Photoshop CS6 (RRID:SCR_014199, Adobe Systems, San Jose, CA, USA) and minimally processed to adjust the brightness, contrast, and background.

### 2.5. Statistics

Statistical analysis and graph plotting were performed in Prism GraphPad version 6.04 for Windows (RRID:SCR_002798, GraphPad Software, San Diego, CA, USA). For bodyweight gain analysis and comparison of the effects of low and high doses of BrdU and EdU, two-way repeated-measures ANOVA, followed by Tukey’s multiple comparisons test, was used. For the comparison of the two groups, Mann–Whitney U-test was used. For the comparison of three or more groups, ordinary one-way ANOVA, followed by Sidak’s or Tukey’s multiple comparisons tests, was used. Statistical differences were considered significant at *p* < 0.05. All data are presented in the graphs as a mean ± standard error of the mean.

## 3. Results

### 3.1. Different Doses of Thymidine Analogs Reveal Equivalent Numbers of Dividing Cells in the Dentate Gyrus

**Figure 1:**
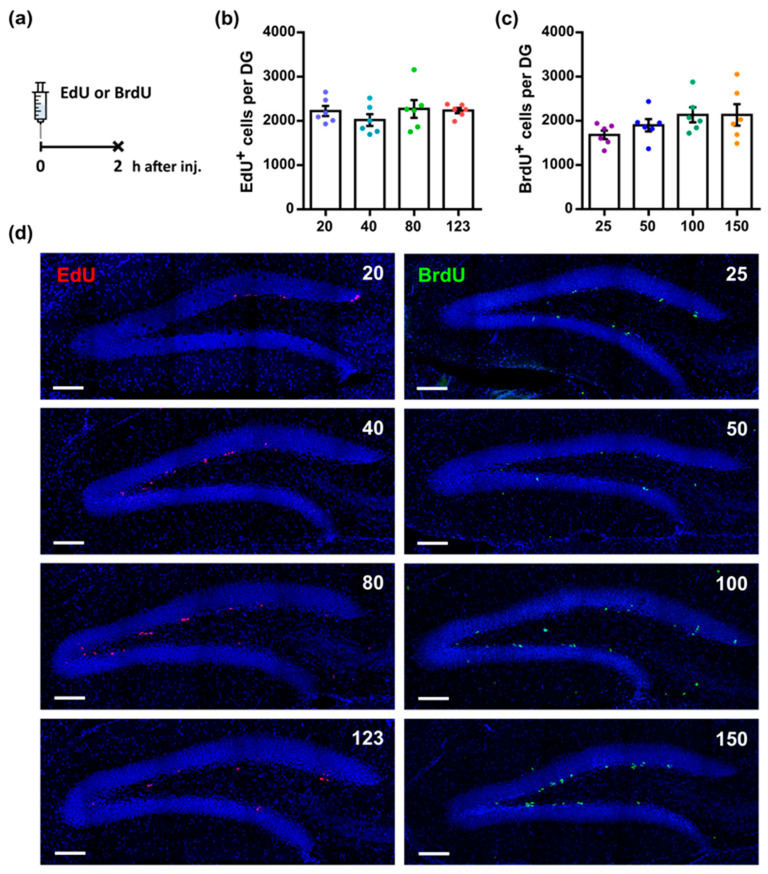
Different doses of thymidine analogs result in equivalent numbers of labeled cells in the SGZ of the DG. (**a**) Scheme of injections; EdU or BrdU at different doses (EdU: 20, 40, 80, or 123 mg/kg; BrdU: 25, 50, 100, or 150 mg/kg) were injected 2 h before euthanasia. (**b**) The number of EdU^+^ cells in the SGZ. (**c**) Number of BrdU^+^ cells in the SGZ. One-way ANOVA did not reveal a significant difference between groups (not marked). (**d**) Representative images of EdU- and BrdU-labeled cells in the sections of the DG; doses are indicated; bar is 100 µm.

We first determined whether different doses of nucleotide analogs label different numbers of proliferating cells in the neurogenic subgranular zone (SGZ) of the DG, if assessed shortly after administration. We injected mice with a wide range of doses of either EdU or BrdU, collected the brains 2 h later, and analyzed the number of EdU- or BrdU-labeled cells in the brain sections (Figure 1a). No statistically significant differences were found in the number of labeled cells in the SGZ in mice receiving EdU at doses of 20, 40, 80, or 123 mg/kg (Figure 1b; 20: 2223 ± 114.7, 40: 2019 ± 133.3, 80: 2270 ± 202.1, 123: 2233 ± 59.3, *p* = 0.5757, one-way ANOVA, and Tukey’s multiple comparisons test). In addition, no significant difference in the number of labeled cells in the SGZ was found among mice receiving different doses of BrdU (Figure 1c; 25: 1681 ± 97.7, 50: 1898 ± 139.8, 100: 2134 ± 169.3, 150: 2134 ± 241.8, *p* = 0.2140, one-way ANOVA, and Tukey’s multiple comparisons test). Two-way ANOVA showed a significant difference in the factor “Analog” but not the factor “Dose” (factor “Analog”: *p* = 0.0471, factor “Dose”: *p* = 0.2108, interaction: *p* = 0.4310); however, Tukey’s multiple comparisons test did not show any difference between groups. Thus, all examined doses of the two analogs, EdU and BrdU, resulted in comparable numbers of labeled cells. Representative images of EdU and BrdU labeling in the DG are shown in Figure 1d.

### 3.2. A Single Injection of EdU Does Not Affect Survival and Subsequent Division of Hippocampal Neural Precursor Cells

**Figure 2:**
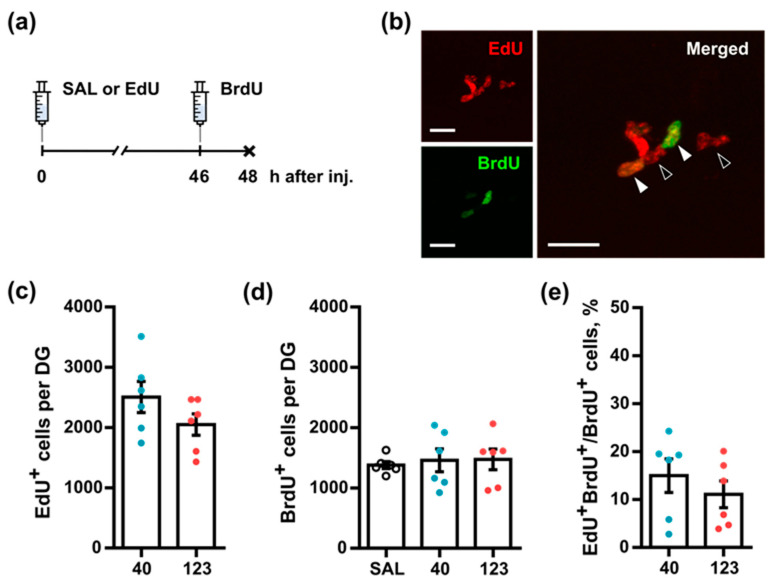
Single injection of EdU does not affect the survival and subsequent division of hippocampal neural precursor cells. (**a**) Scheme of injections. Either saline or EdU at different doses (40 or 123 mg/kg) was injected. BrdU at a dose of 50 mg/kg was injected 46 h later, as well as 2 h before euthanasia. (**b**) Representative images of EdU- and BrdU-labeled cells. Filled arrowheads indicate EdU ^+^ BrdU^+^ cells, and empty arrowheads indicate EdU ^+^ BrdU^-^ cells; the scale bar is 20 µm. (**c**) Number of EdU^+^ cells in the DG. (**d**) Number of BrdU^+^ cells in the DG. (**e**) Number of EdU ^+^ BrdU^+^ double-labeled cells, as a fraction of BrdU^+^ cells. (**c**,**e**) Mann–Whitney U-test, (**d**) one-way ANOVA; no significant difference between groups was found.

To investigate the effects of administered EdU on hippocampal cell division and survival in adult mice, we injected mice with low and high doses of EdU (40 and 123 mg/kg) or saline as a control. Furthermore, for examining the effects of EdU on the subsequent cell divisions, we administered a second thymidine analog, BrdU, 46 h after the injection of EdU and dissected the brains 2 h later (48 h after the first injection, Figure 2a).

Representative images of staining for EdU, BrdU, and the specificity of their colocalization are shown in Figure 2b. The number of EdU-labeled cells in mice that received 123 mg/kg of EdU was similar to that in mice injected with a dose of 40 mg/kg (Figure 2c; *40*: 2506 ± 257.7, *123*: 2049 ± 178.4, *p* = 0.2229, Mann–Whitney U-test). Additionally, EdU incorporation into cells did not alter the subsequent proliferation of cells in the SGZ, as evidenced by the similar number of BrdU-positive cells in the *SAL* and EdU-injected groups (*SAL*: 1378 ± 57.88, *40*: 1459 ± 190.1, *123*: 1473 ± 171.8, *p* = 0.8936, one-way ANOVA (Figure 2d)). In addition, no significant difference in the number of cells labeled with both analogs was found between mice receiving different doses of EdU (Figure 2e; *40*: 15 ± 3.5, *123*: 11 ± 2.8, *p* = 0.4740, Mann–Whitney U-test). Thus, within a span of 2 days, a single injection of EdU at different doses did not affect the subsequent division and survival of the hippocampal neural precursor cells.

### 3.3. EdU and BrdU Have Different Long-Term Effects on Hippocampal Neurogenesis

**Figure 3:**
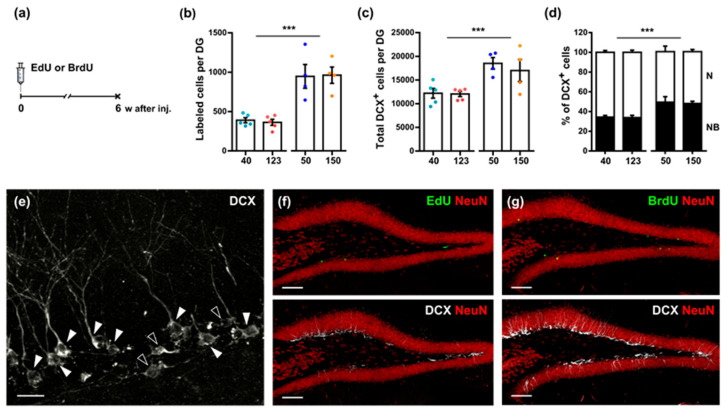
EdU and BrdU have different long-term effects on hippocampal neurogenesis. (**a**) Scheme of injections; mice were euthanized 6 weeks after a single EdU or BrdU injection. (**b**) Number of EdU^+^ (40 and 123 mg/kg) and BrdU^+^ (50 and 150 mg/kg) cells in the DG. (**c**) Total number of DCX^+^ cells in DG. (**d**) The proportion of young neurons (N) and neuroblasts (NB) among DCX^+^ cells. (**e**) Different subtypes of DCX^+^ cells; empty arrowheads indicate neuroblasts (NB) and filled arrowheads indicate young neurons (N), the scale bar is 20 µm. (**f**,**g**) Representative images of EdU^+^, BrdU^+^, and DCX^+^ cells, bar is 100 µm. (**b**–**d**) *** *p* < 0.0005, factor “Analog”, two-way ANOVA.

To examine and compare the long-term effects of various doses of EdU and BrdU on hippocampal neurogenesis of adult mice, we analyzed the numbers of labeled cells in the SGZ 6 weeks after thymidine analog injection (Figure 3a). Both low and high doses of EdU (40 and 123 mg/kg) resulted in a comparable number of labeled cells in the DG (Figure 3b,f; EdU: *40*: 389.7 ± 35.3, *123*: 362.8 ± 38.2, *p* = 0.2460, Mann–Whitney test). Similar findings were obtained for low and high doses (50 and 150 mg/kg) of BrdU (Figure 3b, g; BrdU: *50*: 947.5 ± 150.9, *150*: 962.7 ± 103.6, *p* = 0.9714, Mann–Whitney test). However, two-way ANOVA indicated a significant difference in the factor “Analog” (factor “Analog”: *p* < 0.0001, factor “Dose”: *p* = 0.9461, interaction: *p* = 0.8091). Tukey’s multiple comparison test found a difference between the EdU and BrdU groups. Furthermore, the administration of either low or high doses of EdU or BrdU revealed, for each nucleotide, a comparable number of the DG cells labeled with DCX, which is an early marker of neuronal lineage (Figure 3c,f,g; EdU: *40*: 12,200.0 ± 1031.0, *123*: 12,048.0 ± 537.2, *p* = 0.9444, Mann–Whitney test; BrdU: *50*: 18,502.0 ± 1230.0, *150*: 16,987.0 ± 2312.0, *p* = 0.6571, Mann–Whitney test). However, paralleling the results with nucleotide incorporation, there was a significant difference between the EdU and BrdU groups (two-way ANOVA; factor “Analog”: *p* = 0.0008, factor “Dose”: *p* = 0.5396, interaction: *p* = 0.6151).

DCX-positive cells represent several progenitor subpopulations at various stages of neuronal maturation [19,20,21]. We defined and combined the subclasses of early neuronal progenitors (A–C-type—horizontal cells without apical dendrites) into one category, as previously described [19,20,21], and designated them as neuroblasts (NB in Figure 3d,e). We also categorized and combined more mature cells of the D-G subtypes (with large nuclei and apical dendrite trees) into another category (young neurons or N in Figure 3d,e). Comparison of the relative counts of these two categories of progenitor cells did not indicate a difference between the low and high doses of EdU and BrdU, but found the difference between the analogs (Figure 3d; two-way ANOVA; factor “Analog”: *p* = 0.0004, factor “Dose”: *p* = 0.7793, interaction: *p* = 0.8869): the ratio of NB to N was 34 to 66% in the EdU group and 49 to 51% in the BrdU group.

Thus, our results show that both low and high doses of EdU and of BrdU tagged a comparable number of cells in the adult brain 6 weeks after injection. However, the number of EdU-labeled cells was lower than that of BrdU-labeled cells, thus suggesting that administration of EdU adversely affected cell survival within a 6-week interval. Both thymidine analogs had similar effects on the numbers of less and more mature subpopulations of DCX cells examined 6 weeks after injection. Thus, while higher doses of EdU and BrdU do not show advantage over lower doses, when labeling adult-born neurons in long-term experiments, the usage of BrdU is preferable to that of EdU.

### 3.4. A Single Injection of EdU Affects Somatic Development of Newborn Pups in a Dose-Dependent Manner but Does Not Affect Hippocampal Neurogenesis

**Figure 4:**
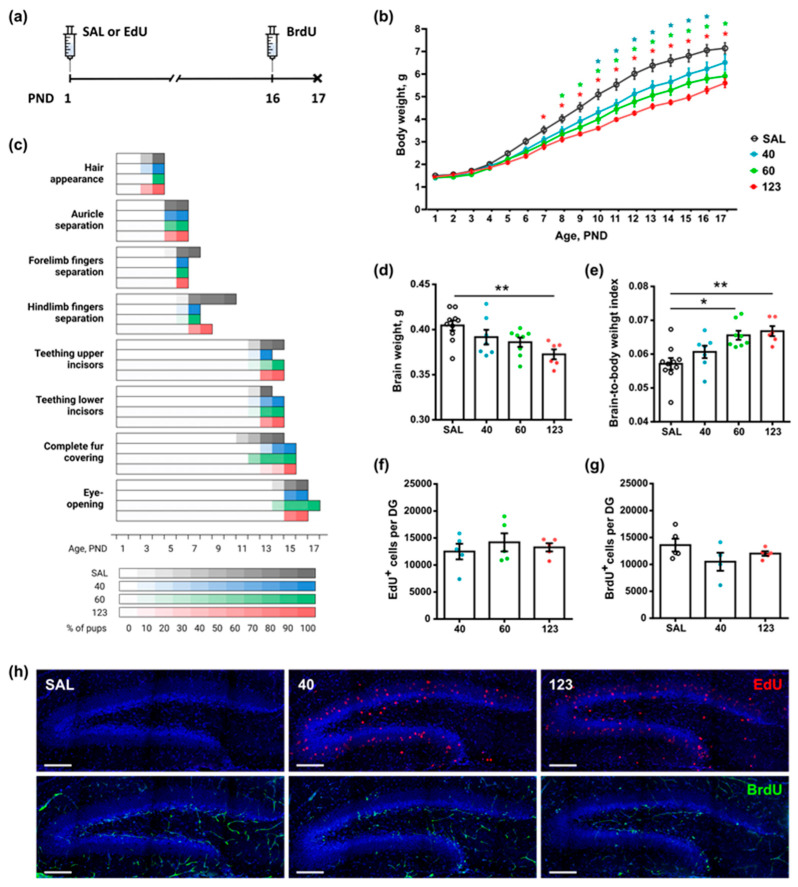
Effects of a single injection of EdU on somatic development and neurogenesis of newborn pups. (**a**) Scheme of injections. EdU at different doses (40, 60, or 123 mg/kg) or saline (SAL) was administered on PND1. BrdU (50 mg/kg) was injected on PND16, and pups were euthanized on PND17. (**b**) Weight change dynamics in control and EdU-injected pups. Two-way ANOVA, Tukey’s posthoc test, * *p* < 0.05. Differences between the *SAL* and EdU groups are shown. Blue, green, and red stars indicate *p*-values for the 40, 60, and 123 mg/kg groups, respectively. (**c**) Somatic development of control and EdU-injected pups. (**d**) Brain weight in PND17 pups. (**e**) Brain weight to whole bodyweight ratio in PND17 pups. (**f**) Number of EdU^+^ cells in the DG in PND17 pups (16 days after single injection). (**g**) Number of BrdU^+^ cells in the DG in PND17 pups (24 h after single injection). (**d**–**g**) One-way ANOVA, Sidak’s posthoc test, * *p* < 0.05, and ** *p* < 0.01. (**h**) Representative images of EdU- and BrdU-labeled cells; the scale bar is 100 µm.

To examine the effects of EdU on early postnatal development, we followed somatic development and neurogenesis in newborn mice. We administered EdU at doses of 40, 60, or 123 mg/kg or with saline as a control, through a single injection to pups on PND1 and injected BrdU on PND16. We then monitored eight critical morphometric features, including incisor teething, ear and finger separation, fur appearance, eye opening, and bodyweight daily, until PND17, when the mice were euthanized (Figure 4a).

The analysis of the weight gain dynamics of the newborn pups did not reveal any differences among the four groups until PND6 (Figure 4b). However, starting from PND7, a significant lag in bodyweight gain was observed in the pups receiving EdU at a dose of 123 mg/kg. Significant differences between the *123* and *SAL* groups persisted for the following days of postnatal development. Beginning from PND8, we also found a lag in bodyweight gain in pups receiving 60 mg/kg of EdU, which was also evident until PND17. The dose of 40 mg/kg significantly influenced bodyweight from PND 10 to 16; however, on PND17, no significant difference in bodyweight was observed between the *40* and *SAL* groups. We also found a significant difference in bodyweight between groups *123* and *40* starting from PND12: the bodyweights of animals receiving a higher dose were lower (*p* = 0.01, Tukey’s multiple comparisons). Thus, none of the examined doses of EdU affected the bodyweight of newborn mice in the first postnatal week. However, a lag in bodyweight gain between the control and EdU-injected mice was apparent from the second week of life (PND7–PND10) until the end of measurements (PND17) (Figure 4b).

Together, these experiments showed that the higher the dose of EdU received at birth, the slower the bodyweight gain and earlier the appearance of a difference in bodyweight between EdU-injected and control animals. However, these differences were evident only starting from the second week of postnatal development.

We next assessed the potential effects of EdU on perinatal somatic development by determining the percentage of animals with fully developed features each day between PND1 and PND17 (Figure 4c). We found no differences in the characteristics manifested during the first postnatal week between the control and EdU-injected pups. All studied animals were fed each day (on the basis of the sign of milk in the stomach, which is discernible in the first week of postnatal development); primary hair cover appeared by PND4, and separation of auricles was evident by PND6. Later, differences in somatic development between the EdU-injected and control pups began to appear (Figure 4c). The forelimb finger separation in pups of the *SAL* group was completed by PND7, but evident one day earlier, by PND6, in all pups receiving EdU. The hindlimb finger separation in the EdU-injected pups also exceeded that of the control mice (PND10 in *SAL*, PND8 in *123*, and PND7 in *40* and *60*). The upper incisor teething was completed by PND14 in the *SAL*, *60*, and *123* groups, but occurred one day earlier in pups in the *40* group. In contrast, two other features—lower incisors teething and complete fur covering—were evident 1 day earlier in the control mice than in the EdU-injected mice. Finally, eye opening in all groups, except *60*, was completed by PND16. In mice in the *60* group, both eyes opened 1 day later, on PND17 (Figure 4c).

Thus, the administration of EdU did not affect the somatic features that are fully formed within the first week of development. However, the features that mature during the second week were affected, with differences being evident earlier or later in the experimental groups than in the control pups.

Next, we compared the brain weight and brain-to-bodyweight index of the pups. The brain weight was similar in mice in the *SAL*, *40*, and *60* groups (0.40 ± 0.006, 0.39 ± 0.008, and 0.39 ± 0.005 g, respectively); however, in the *123* pups, it was significantly lower than that of the control *SAL* group. The ratio of brain weight to bodyweight was similar in the *SAL* and *40* groups, but significantly higher in the *60* and *123* groups.

Thus, lower doses of EdU, i.e., 40 mg/kg, did not noticeably affect brain weight. At the same time, the higher the dose of EdU received at birth, the more pronounced the negative effect on brain development was.

We also examined the effects of EdU administered at birth on hippocampal neurogenesis at PND17. We analyzed the numbers of EdU- and BrdU-labeled cells, with BrdU injected one day before the analysis. EdU^+^ cells representing the newborn neuron population were widely distributed in the granule cell layer and SGZ of DG (Figure 4h); they had the large, rounded nuclei characteristic of neurons. The numbers of EdU^+^ cells were similar in the mice of all four groups (Figure 4f; *40*: 12,497.0 ± 1444.0, *60*: 14,198.0 ± 1673.0, and *123*: 13,274.0 ± 758.7), with no significant differences between the *40*, *60*, and *123* groups (*p* = 0.6798, one-way ANOVA, Sidak’s multiple comparisons test). The BrdU-labeled cells were located primarily in the SGZ and corresponded to dividing stem and progenitor cells. The numbers of BrdU^+^ cells in the *SAL*, *40*, and *123* groups were also similar (Figure 4g; *SAL*: 13,584.0 ± 1185.0, *40*: 10,494.0 ± 1679.0, and *123*: 12,014.0 ± 423.1), without significant differences between the *SAL*, *40*, and *123* groups (*p* = 0. 2121, one-way ANOVA, Sidak’s multiple comparisons test).

Thus, although a high dose of EdU (123 mg/kg) negatively affected bodyweight gain and brain weight in the mouse pups, it did not affect hippocampal neurogenesis, as assessed in 2.5 weeks old mice.

## 4. Discussion

Here, we sought to assess the efficacy and possible adverse effects of the widely-used thymidine analog markers of proliferating cells, as presented in the context of neurogenesis and perinatal development. We focused on BrdU and EDU—two analogs that are used most widely and revealed by different approaches—with an antibody for BrdU and fluorescent azide for EdU. We examined the efficacy of BrdU and EdU for tagging neural stem and progenitor cells, as well as their progeny, in the adult mouse hippocampus, a setting in which they are used particularly often [22,23,24,25]. We also investigated the effect of various doses of EdU, administered once at birth, on the subsequent early somatic development of the mouse pups.

Our results demonstrate that both analogs can be effectively used in various experimental designs in a wide range of doses. Our results also indicate that EdU, at doses frequently used to tag dividing cells, can affect early postnatal development and the long-term survival of the labeled cells in the adult brain. Together, our results help dispel several misconceptions about using the thymidine analogs for labeling dividing brain cells and suggest practical recommendations for their application.

Regarding adult hippocampal neurogenesis, we evaluated the saturation doses of BrdU and EdU, as assessed two hours, two days, or six weeks after administration. We found that, within a wide (six-fold, from 20–25 to 120–150 mg/kg) range of administered doses, BrdU and EdU mark the same number of cells in the DG two hours after administration, both within and between the BrdU and EdU groups (Figure 1). Importantly, in this short time interval, tagged neural stem and progenitor cells have not yet divided or been eliminated, i.e., their number has not been altered by duplication or death, inherent toxicity or suppression of DNA replication by BrdU or EdU, or label dilution, which accompanies the segregation of labeled chromosomes into two daughter cells. These numbers are also similar to those obtained with iodo- or chloro-derivates of thymidine or endogenous markers of cell cycling, such as Ki67 or PCNA [6,8,9,18] (and our unpublished observations). Together, these results indicate that, for the adult mice, the initial efficiency of labeling is tolerant to a wide range of the administered EdU or BrdU doses.

When assessing the pool of the labeled cells in the DG two days after a single administration of high (123 mg/kg) or low (40 mg/kg) doses of EdU, we again detected a similar number of cells (Figure 2c). Moreover, when the cells that were duplicating their DNA at that time were tagged using a pulse injection of BrdU shortly before the analysis, the number of BrdU-labeled cells did not differ for mice that received either dose of EdU or saline two days earlier (Figure 2c–e), suggesting that, within this time interval, EdU does not notably interfere with cell division in the DG. The number also did not differ for the population of the EdU^+^ BrdU^+^ double-labeled cells, arguing against the suppression of division of cells tagged with EdU two days earlier or changes in the length of the cell cycle or the S phase of those cells. Together, these results suggest that, in the course of two days (approximately two cell cycles of dividing hippocampus progenitor cells [8,26,27]) and within the 20–120 mg/kg range of doses, EdU does not perturb cell proliferation, increase the number of cells undergoing programmed elimination, or induce cell damage/death.

When assessed 6 weeks after administration, there was, again, no evident difference between the effects of high vs. low doses of EdU or BrdU, in regard to the number of labeled cells (most of them corresponding to young neurons) or less mature and more mature DCX-positive neuroblasts in the DG (Figure 3). However, for each examined dose, there was a significant 2.5-fold difference in the number of EdU- and BrdU-labeled cells per DG (Figure 3b) and 1.5-fold difference in the number of DCX-positive cells (Figure 3c). Since there was no evidence of EdU affecting cell proliferation or survival two hours or two days after administration (Figure 1 and Figure 2), these results may indicate that EdU incorporation in the genome specifically negatively impacts the long-term survival of the labeled (presumably postmitotic) cells. Additionally, EdU administration shifts the ratio of neuroblasts to young neurons towards young neurons by reducing the fraction of neuroblasts, but not neurons, among DCX-positive cells.

Overall, our results indicate that low doses of EdU and BrdU (up to 20–25 mg/kg) are sufficient for labeling the neural progenitor cells. This stands in contrast to some of the previous findings, which indicate a pronounced dependence of the number of labeled cells in the adult neurogenic zones on the doses of administered analogs; for instance, several reports have indicated that doses of 150–200 mg/kg of BrdU are saturated for the purpose of following adult hippocampal neurogenesis [26,27,28,29]—for review, see [1,2]. The differences in the studies’ findings might potentially be due to the improved methods of detection and their specificity and sensitivity, increased sensitivity of the detection protocols, affinity of antibodies, quantum yield of the fluorescent label, and other methodological details. However, we also note that low concentrations of analogs may be insufficient for studying cells that undergo multiple rounds of divisions, each of which decrease the signal intensity per cell.

Regarding somatic development, we examined whether EdU can affect some of the critical milestones of the perinatal development of the mouse pups. We found that a single injection of EdU to newborn pups affects the course of their somatic development, including brain development. Within the first postnatal week, neither dose of EdU (40, 60, or 123 mg/kg) had a significant effect on the weight (Figure 4b) or somatic maturation (Figure 4c) of the pups. However, starting with PND7, the effect of the 123 mg/kg dose of injected EdU on the weight of the animals became evident. Starting with PND8, the effect of the 60 mg/kg dose of EdU became evident; finally, from PND10, the effect of the lower dose (40 mg/kg) also became apparent. The high dose of EdU also decreased the brain weight, as evaluated at PND17 (Figure 4d). Thus, EdU has a negative effect on early postnatal development in mice, as evidenced by a dose-dependent decrease in the rates of body weight gain and brain weight.

EdU, at all examined doses, either did not appreciably affect the parameters of the pups’ somatic development, such as hair appearance and auricle separation, or slightly delayed their features, such as teething of the lower incisors and complete fur covering (Figure 4c). Intriguingly, the strongest effects were observed on fore- and hindlimb finger separation, a feature accelerated by 1–3 days. Conceivably, EdU may promote apoptosis of the cells of the interdigital tissue, thus accelerating the complete disappearance of webbing in the perinatal limbs. EdU also affected the brain weight of the pups when administered at a high (123 mg/kg) dose, although not the number of surviving (EdU-labeled) or dividing (BrdU-labeled) cells in the DG (Figure 4d–g).

Together, our results convey a multifaceted message regarding the possible adverse effects of EdU and BrdU on cell division, differentiation, and survival, as well as tissue development. In the juvenile mice, high doses of EdU altered several morphometric and somatic indices of general postnatal development, such as bodyweight during the second week of life, brain weight, finger separation, teething, or fur covering (Figure 4a–e). However, they did not affect DG neurogenesis or neural progenitors’ survival during that period (Figure 4f,g). In the adult brain, all of the examined doses of EdU and BrdU were equally effective in labeling the dividing cells. However, even at lower doses, EdU may affect the long-term survival of the labeled cells.

EdU, and to a lesser degree, BrdU can potentially induce cytotoxic and genotoxic stress [30,31,32,33,34,35,36,37,38,39] (note that other modified alkene derivatives, such as 5-vinyl-2′-deoxyuridine [11] or (2′S)-2′-deoxy-2′-fluoro-5-ethynyluridine [12], may show less toxicity in vitro and in vivo). It may stall the replication fork, induce inter-strand cross-links and double-strand breaks, suppress G2-M transition, increase the frequency of endoreplication, and induce sister chromosome exchange and chromosome aberrations [1,30] (notably, the latter is manifested only in the second post-labeling metaphase [30]). However, EdU also activates DNA repair systems, with the DNA damage eventually repaired by homologous recombination (and, to a lesser degree, by non-homologous end-joining repair). These observations are compatible with our findings of adverse effects of EdU in the adult DG six weeks after administration, but not immediately after administration or during the two post-labeling days, when the repair systems might be mitigating the genotoxic effects of nucleotide analogs.

Notably, the current knowledge regarding the effects of EdU and BrdU has been largely derived from experiments with cells in culture. Our study is one of the few [35,38,39,40,41,42] attempting to evaluate the potential adverse effects of EdU and BrdU in the live animal. Our findings may, therefore, be relevant to the possible mechanisms of repair of analog-induced aberrations, integrity of the blood-brain barrier (which is less mature during the first postnatal weeks), actual bioavailability of the nucleotides in the brain (including the uptake and degradation of nucleotides), and maintenance of the cellular pool of thymidine and other nucleotides. Our results suggest that the adult brain may be better equipped than the juvenile brain to mitigate the harmful effects of thymidine analogs and, at least at lower concentrations, EdU and BrdU can be used as reliable markers of cell division and fate.

Our study provides practical suggestions for using EdU and BrdU to investigate neurogenesis and development. Nucleotide dosing must strike a balance between efficiently tagging dividing and differentiating cells, thus preserving the label (even after rounds of cell and DNA duplication) and avoiding toxicity.

- If the goal of the experiment is to determine the number of neural progenitor cells engaged in the cell cycle, low doses of either EdU or BrdU (up to 20 mg/kg) are almost as effective as the higher doses (up to 120–150 mg/kg).
- If the goal is to investigate cells that may have undergone several rounds of division (and, therefore, dilution of the initial label), higher doses (120–150 mg/kg of EdU or BrdU) may be preferable.
- If the goal is to investigate the long-term changes, e.g., survival and differentiation, in cells that were tagged 1–3 months earlier, using BrdU may be preferable to EdU.
- For cell labeling with multiple markers, it is safer to use EdU as the last label.
- For labeling cells in the perinatal animals, it may be important to take into account that EdU may have an adverse effect on several developmental milestones; at the same time, administration of EdU does not have a noticeable impact on hippocampal neurogenesis in juveniles.

In summary, our results indicate that, although high doses of EdU can affect early postnatal somatic development and long-term cells survival of cells in the brain, both EdU and BrdU, with precautions, can be effectively used in a wide range of doses to investigate perinatal and adult neurogenesis.

## Data Availability

Data is contained within the article.
